# Supplementary material for: The Construction and Exploration of a Comprehensive MicroRNA Centered Regulatory Network in Foxtail Millet (Setaria italica L.)
Source: Front Plant Sci. 2022 May 6;13:848474. doi: 10.3389/fpls.2022.848474 (PMC9121102; doi:10.3389/fpls.2022.848474)
Supplement: Supplementary file 2 [file Data_Sheet_2.zip › Sit-miR171f_Seita.3G064300.1_1469_TPlot.pdf]

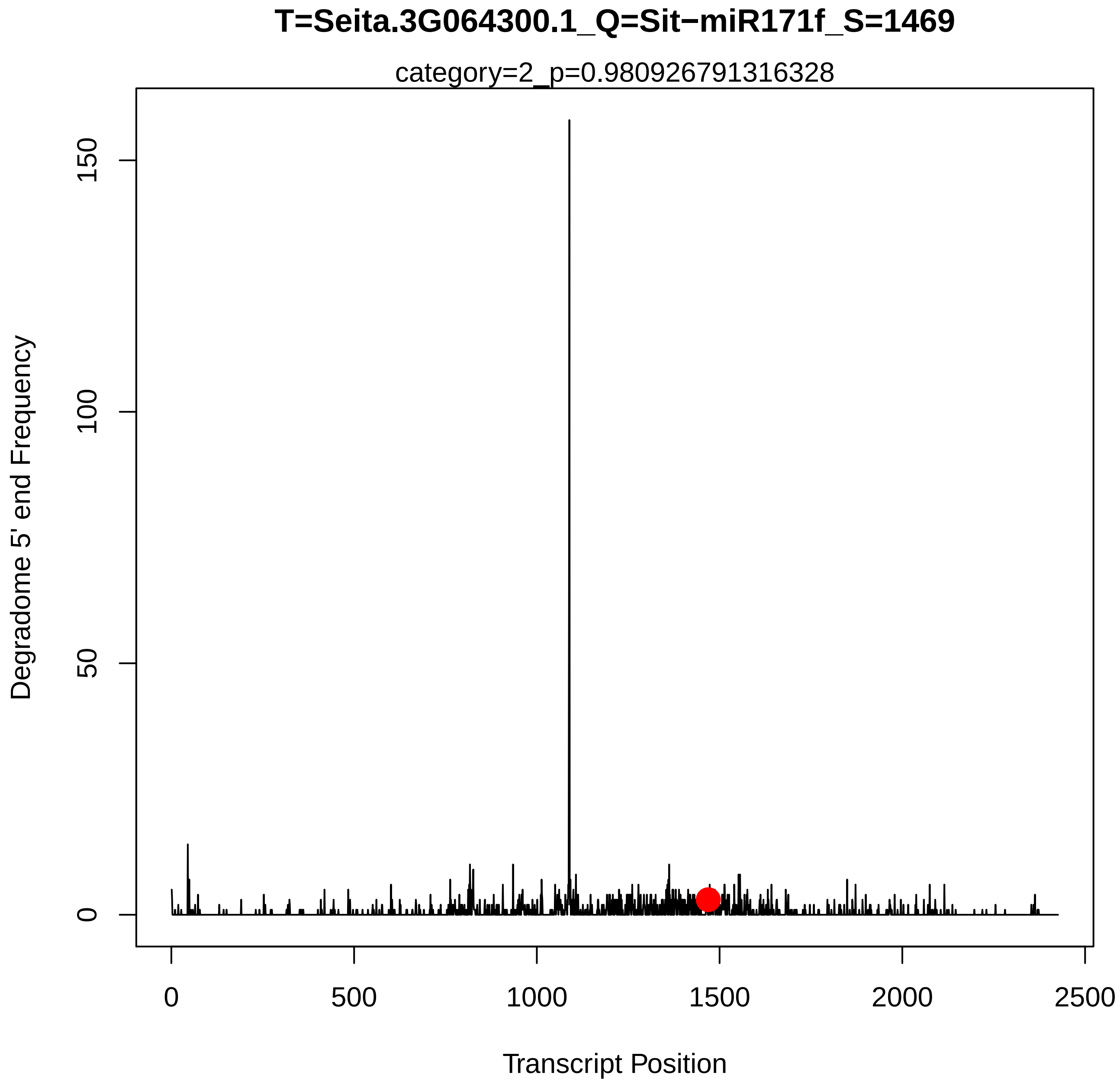

T=Seita.3G064300.1_Q=Sit−miR171f_S=1469
category=2_p=0.980926791316328
Degradome 5' end Frequency
0
50
100
150
0
500
1000
1500
2000
2500
Transcript Position
